# “Turn Around and Forget”: Assessment of the Cognitive Inhibitory Effect of Working Memory Information Using the List-Before-Last Paradigm

**DOI:** 10.3389/fpsyg.2018.02516

**Published:** 2018-12-17

**Authors:** Xiaojun Zhao, Changhao Liu, Changxiu Shi

**Affiliations:** School of Education, Hebei University, Baoding, China

**Keywords:** list-before-last paradigm, working memory, cognitive inhibitory effect, the nature of the signified information, the number of retrieval cues

## Abstract

This study mainly discusses whether the cognitive inhibitory effect of working memory information is affected by the nature of the signified information and the number of retrieval cues in the inhibitory information. Experiment 1 of our study examined the effect of concreteness on the information retrieval phase under different cognitive inhibition scenarios that were distinguished by the nature of the signified information and the number of retrieval cues in the inhibitory information. Experiment 2 of our study examined the effect of the number of retrieval cues in the inhibitory information on the cognitive inhibitory effect under different cognitive inhibition scenarios. The results of both experiments showed that information displaying more concrete characteristics exerted a greater the cognitive inhibitory effect during the working memory task, and a greater cognitive inhibitory effect was produced when all of inhibition retrieval information clues are provided than when none of the clues are provided in the working memory task. Based on these results, the concreteness effect on cognitive inhibition exists, and when all retrieval clues for inhibitory information are provided, the cognitive inhibitory effect might be greater.

## Introduction

Imagine a situation in which we cannot find something but would like to try to remember concrete information to answer the question “Where did I last see it?” At this point, our memory system begins to function. According to the theory of information processing, our memory system initially encodes and stores concrete information and then helps us retrieve this information when needed (Howard and Kahana, 2002; Lehman and Malmberg, 2009). Fortunately, we are able to retrieve concrete information with the help of our memory system. However, we may fail to retrieve the information in some cases: “I just saw it, but I can’t remember where it is.” Therefore, even though we do not forget certain information for a long time, why do we forget? This question is the one we wished to solve in the present study; namely, what is the mechanism underlying the “forgetfulness” of working memory in the information retrieval stage, or what is the mechanism of “cognitive inhibition”?

The term “cognitive inhibition” in this study refers to the internal process in which an individual inhibits the retrieval of irrelevant information and maintains the information relevant to the task in working memory (Harnishfeger and Bjorklund, 1993).

### The Theoretical Basis of the Cognitive Inhibitory Effect: Context Change Model and Temporal Context Model (TCM)

#### Context Change Model

First, the reason why we study working memory is to eliminate the interference of normal forgetting and better explain the abnormal forgetting phenomenon of “turn around and forget.” Second, we describe the “forgetting mechanism” as “cognitive inhibition mechanism” because our experiments are based on the “context-change model,” and this assumption is in fact a type of cognitive inhibition theory (Sahakyan and Kelley, 2002). Furthermore, the context change model assumes that different memory tasks lead to changes in people’s internal situations, and the inconsistency between encoding and retrieval leads to cognitive inhibition (forgetting). Thus, the phenomenon of “turn around and forget” may be related to a change in our memory, and its nature may be “a cognitive inhibitory effect” on concrete working memory information.

#### Temporal Context Model (TCM)

According to a previous study (Sahakyan and Hendricks, 2012), the mechanism of individual context change situations may utilize a temporal context model (TCM). This theory postulates that each new unit of information processing will cause a change in the cognitive environment, namely, in our information processing ability. Therefore, our internal psychological status will also constantly change throughout the experiment. In addition, the successful retrieval of information may lead to a psychological context change. The TCM emphasizes that information processing occurs in a particular order and hypothesizes that information obtained later might affect information obtained earlier during memory and retrieval processes.

Although the TCM has not yet been supported by a large number of experiments, it offers a new concept while we research the cognitive inhibitory effect of working memory information. In the information retrieval phase, greater retrieval of irrelevant information about the task produces greater inhibitory effects and increases the difficulty in retrieving information relevant to the task.

### Paradigm of Experimental Research on Cognitive Inhibition: The List-Before-Last Paradigm

#### The List-Before-Last Paradigm and Its Limitations

Our experiment uses the list-before-last paradigm to examine the context change during the cognitive inhibition process. This paradigm has been reported to be a reliable test of an individual’s context change (Shiffrin, 1970; Jang and Huber, 2008; Sahakyan and Hendricks, 2012; Sahakyan and Smith, 2014). During the experiment, participants will first be distinguished according to the different types of memory tasks (e.g., restudy L1 task and retrieval L1 task). Then, they will sequentially memorize three lists (L1, L2, and L3), each of which contains a specific number of words. However, after memorizing L1 and L2, participants will memorize L1 again using different strategies, according to the different types of memory tasks we established. When participants have memorized all lists, they will be asked to provide free recall of L2 as the final test. Instigated by the different methods for retrieving L1, the contextual similarity/continuity between the adjacent lists (L2 and L3) was disrupted (what we called “context-change” in our study). Furthermore, reinstating the L2 context will become more difficult for participants because context drifted from one list to the next list in a somewhat gradual fashion and was disrupted by the retrieval of L1. Therefore, using this paradigm, we examined the degree of the interruption effect (caused by the retrieval of L1 using different strategies) by testing the effects of the L2 context during the final test. Greater free recall of the L2 context represents a smaller interruption effect of the retrieval of L1. This “interruption effect of the retrieval of L1” is defined as “the cognitive effect” in the present study. Meanwhile, in the list-before-last paradigm, we consider L1 as “inhibitory information” and L2 as “inhibited information.”

However, previous studies have created cognitive inhibition situations by destroying the gradual context drifts through different L1 retrieval tasks, and an individual’s context change also initially occurred during different cognitive inhibition tests (Sahakyan and Hendricks, 2012). However, we have not yet identified an experimental operational index to measure the extent of that context change. Therefore, we aim to solve this problem in the present study.

#### Localization of the List-Before-Last Paradigm

The experimental research on “Chinese words” as the experimental material in the list-before-last paradigm is insufficient. However, some research can help researchers localize this experimental paradigm. In the field of cognitive psychology, studies of “Chinese lexical information processing and storage methods” indicated that the method used to divide words into concrete words (such as 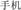
—mobile phone; 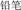
—pencil) and abstract words (such as 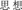
—ideology; 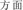
—division) is more mature than the other methods of classifying words because it will help the “Chinese processing context” become more pertinent to the “English processing context”; namely, when the memory and understanding of a same concrete word or an abstract word are employed, the meaning will be clearer and more precise for both Chinese and English speakers than the use of other word classification methods (Sui et al., 2016). This finding provides new insights into the suitability of using the list-before-last paradigm for Chinese words. And Chinese experimental processing situations may be more consistent with the original English experimental processing situations through this word classification method.

Additionally, previous studies have used three different lists of words containing 12 nouns per list (Sahakyan and Hendricks, 2012; Sahakyan and Smith, 2014). However, in a “directed forgetting” study, where participants memorized and recalled two different lists (A and B) of words containing 12 nouns per list in order (A–B), the first four words of list B have the highest recall rate among all words in list B (Pastötter et al., 2012). Thus, during the memorization and recall of a list of words, the semantic information of the first four words is the most accurate for the participants. And depending on this conclusion, the disruption of contextual continuity between adjacent lists (context change) and the cognitive inhibitory effect caused by the L1 retrieval task may be more likely reflected in the first four words of L2 in the list-before-last paradigm. When the number of words in each list was reduced to four in the list-before-last paradigm, maybe most of the words could process equally by participants throughout the experiment and the serial position effect (SPE) with more memory load in each list could avoid partly. Accordingly, the opportunity of each item of L2 to be recalled first in the final test was approximately equal. And the disruption of contextual continuity between neighboring lists will indeed inhibit L2. If this hypothesis is true, according to context change model, L2 words will be more difficult to recall in the free recall task (Task 1); and according to the TCM, more L2 words will be recited out-of-order among all three lists of words in the memory sorting task (Task 2). And as more position units changed, a larger inhibitory effect caused by the later information (retrieval L1 task) was observed.

### Factors Influencing the Cognitive Inhibitory Effect: Types of Memory Tasks and Types of Memory Materials

#### Different Memory Tasks Based on Different Numbers of Retrieval Cues for Inhibitory Information

In the presence of different numbers of retrieval cues for inhibitory information (L1), this inhibitory information produces varying degrees of context change (Sahakyan and Smith, 2014). Specifically, in the list-before-last paradigm, tests in which all retrieval cues for inhibitory information are provided (task of “restudy L1”) cause greater free recall of L2 than tests in which a portion of the clues are provided (task of “retrieval L1”). Thus, a smaller context change may be observed in participants who are provided with all retrieval cues for inhibitory information than in participants who are provided a portion of the clues. Using this strategy, our research should consider the number of retrieval cues for inhibitory information as a factor that may affect the cognitive inhibition of working memory.

#### The Concreteness Effect Is Based on the Processing of Material Referring to Something of a Different Nature

The processing of words that refers to the different nature of things will be affected by the “concreteness effect.” Processing of concrete words is more accurate and faster than processing of abstract words, particularly when words are presented separately (James, 1975; Schwanenflugel and Shoben, 1983; Kroll and Merves, 1986; Bleasdale, 1987; Schwanenflugel et al., 1988). An ERP study focused on explaining the mechanism of the concreteness effect showed that the processing of concrete words evoked greater N400 (N400 is associated with semantic processing) than the processing of abstract words, indicating that the processing of concrete words in the information processing phase may activate more semantic information than the processing of abstract words (Kounios and Holcomb, 1994). Therefore, the degree of change in a mental situation will be greater when an individual is processing concrete words than when an individual is processing abstract words. Thus, in the list-before-last paradigm, the interruption of the context of L2 to L3 concrete words context (by L1 retrieval tasks) may activate more semantic information than abstract words, which also represents a high degree of context change. Under the same interruption condition, a greater amount of inhibition of the semantic information in L1 would occur in the concrete words group than in the abstract words group. According to our former hypothesis, a high degree of cognitive inhibitory effect will be observed with a high degree of context change, which will make the concrete words more difficult to retrieve. Does this “concreteness effect” exist in the information retrieval phase? We have not been able to determine a unified answer from the existing studies. We will explore this question in the present study.

### Research Purpose and Hypothesis Examined in This Study

We aimed to explore the cognitive inhibition mechanism of working memory in the information retrieval phase. In this study, we conducted two experiments to address whether different memory tasks, which are based on different numbers of retrieval cues for inhibitory information, influence the retrieved results. We hypothesized that the task “restudy L1,” which contains large amounts of retrieval cues for inhibitory information in the process of information processing, would produce a smaller cognitive inhibitory effect than the task “retrieval L1,” which contains fewer retrieval cues for inhibitory information in the process of information processing. In the experiment, a greater number of free recall L2 words was presented to the restudy group than to the retrieval group.

We aimed to explore whether the “concreteness effect” existed in the information retrieval phase. We hypothesized that concrete words would cause larger cognitive inhibitory effects than abstract words. In the experiment, more free recall L2 words were presented to the abstract word group than to the concrete word group.

## Experiment 1

In Experiment 1, the cognitive inhibition mechanism of concrete words and abstract words in different memory tasks was investigated under the condition of stimulus-alone-appear. The experiment consists of two tasks. Task 1, “free recall L2 (the inhibited information)”, aims to test the presence of the concreteness effect on the working memory information in the retrieval phase and the different cognitive inhibition modes based on the different numbers of retrieval cues for inhibitory information that will exert different cognitive inhibitory effects. Task 2, “memory sorting L2 (the inhibited information),” aims to test information processing in working memory that will lead to a psychological context change; this “context change” was statistically and simultaneously managed. Furthermore, the logical relationship between these two tasks is described below. After Task 1 confirms the concreteness effect and the cognitive inhibitory effect on the retrieval of working memory information, Task 2 will prove that the mechanism of cognitive inhibition is consistent with the context change model. The participants must complete Task 1 first (time is 60 s) and then complete Task 2 (time is 120 s). The entire experiment lasted approximately 260 s.

Before the experiment, the first four words of each separate list in the experiment were effectively and accurately equal to take advantage of the participants’ memories, and to test the context change, we will control the working memory capacity of the participants using the Operation Span (OSPAN) task (Turner and Engle, 1989). This method has been reported to display high correlation and reliability in measuring the working memory capacity of individuals (Klein and Fiss, 1999; Conway et al., 2005). In this test, we used words to replace letter strings, enabling the test be more similar to the experiment. In the test, participants were required to first determine whether a math equation was correct and then memorize the word according to the math equation. The operation string, for example, might be “(9÷3)–2 = 2? Uncle.” As the number of operation strings gradually increases, the number of words correctly recalled by the participants will comprise their working memory span. However, in our study, we directly established two conditions to improve efficiency: “2 operation strings” for practice and “4 operation strings” in the experiment. All participants were required to report all 4 words, and the correct rate was 100%. All strings were presented sequentially in a random order using the E-prime2.0 software. Each string (in white bold typeface, font size 48 points) was presented for 4 s, and the time interval between the strings was 1 s. The string was presented in the middle of the screen against a black background.

After the experiment, we used a Likert scale (from 1, “when you see the word, you feel very sad,” to 7, “when you see the word, you feel very happy”) to control for the emotional valence of all of the experimental materials and avoid the potential interference from differences in the participants’ emotional valences of the experimental material on the experimental results. All participants were required to complete the scale after the experiment and evaluate those words they observed in the experiment. The results were concrete word, *M* = 4.2733, and abstract word, *M* = 4.2656. The difference was not significant.

### Methods

#### Participants

Sixty volunteers from Hebei University participated in the study. Participants ranged in age from 18 to 24 years (*M* = 21.08, *SD* = 2.04), were not color blind and had normal vision or normal corrected vision. In Experiment 1, participants were randomly assigned to one of four experimental groups: abstract word retrieval, L1 group; abstract word restudy, L1 group; concrete word retrieval, L1 group; and concrete word restudy, L1 group. Each group consisted of 15 participants. After the experiment, each person was rewarded with a gift (stationery, such as pens or notebooks).

#### Materials

First, we identified 18 concrete words (0.0072 average frequency, 17 average strokes) and 18 abstract words (0.0097 average frequency, 16.5 average strokes) from “the most frequently used 3000 words” in *Modern Chinese Frequency Dictionary.* All words were double syllable nouns. Second, all words were randomly assigned to a 6 × 6 format, and 10 non-psychology students (who did not participate in either of the two studies) assessed the concreteness of each word using a Likert scale (from 1, “the word is concrete,” to 7, “the word is abstract”). Before assessing the words, these studies were told that “the word is concrete,” which meant that “the word expresses a concrete image and can also be touched,” and that 1 to 7 points indicated that the word’s concreteness was able to be gradually enhanced. The final results were as follows: concrete word, *M* = 6.2722, *SD* = 0.8641; and abstract word, *M* = 2.0444, *SD* = 0.7155. The differences between the two categories was significant (*p* < 0.01). In the formal experiment, L1, L2, and L3 each contained 4 words, and the other words were used in the practice experiment, with 2 words per list. All words were presented randomly using the E-prime2.0 software. Each word (in white bold typeface, font size 48 points) appeared in the middle of a black background screen for 4 s. The time interval between the words was 1 s.

**FIGURE 1:**
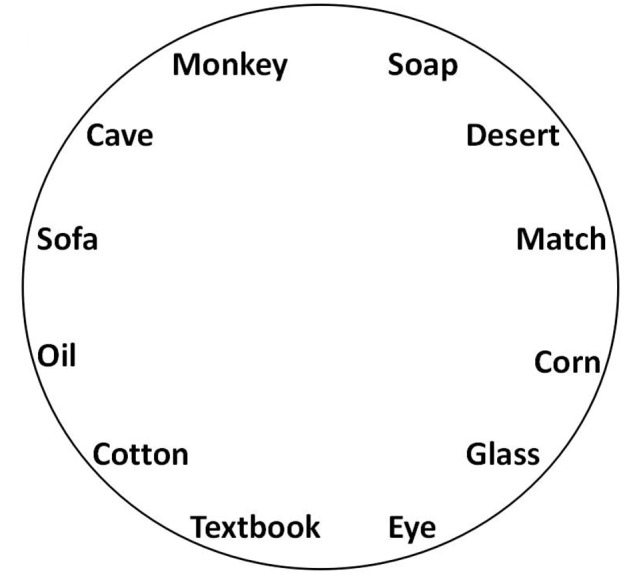
The example of “Memory sorting L2 test paper.”

In Task 2, a “Memory sorting L2 test paper” (Figure 1) was used to avoid a SPE that may occur when information is retrieved. In this test paper, all words were randomly placed in a “circle,” and when the participants finished Task 2, they were required to label the memorized sequence of each word on the test paper.

#### Design

The study used a 2 memory material (concrete words/abstract words) × 2 memory task (retrieval L1/restudy L1) between-subjects design. In Task 1, the dependent variable is the amount of retrieved inhibitory information (L2) and its statistical indicators represent the amount of free recall of L2. In Task 2, the dependent variable is the position change amount of inhibitory information (L2) in the information retrieval phase. Its statistical indicator is the number of position change units, which represents the degree of context change of inhibitory information (L2) in the information retrieval phase. In the present study, “a context-change unit” indicates that every difference in the recall order of a word compared with the original presentation order of each word will be recorded as a context change unit.

#### Procedure

For the retrieval groups, we first presented the following instructions on the screen: “This experiment aims to study our memory. It is divided into three lists. You must memorize all lists, and each list is separated by a plus sign ‘+’. A green plus sign means ‘Continue the memory task,’ and a red plus means ‘Please restudy L1 based on the clue.’ When you are ready, press the space bar to begin.” Then, we presented L1, a green plus sign for 1 s, L2, a red plus sign for 1 s, L1 (each word had only the first word, such as “
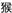
”), and L3. Finally, we presented the instruction, “The experiment is over.” Then, the participants first completed Task 1 followed by Task 2.

**FIGURE 2:**
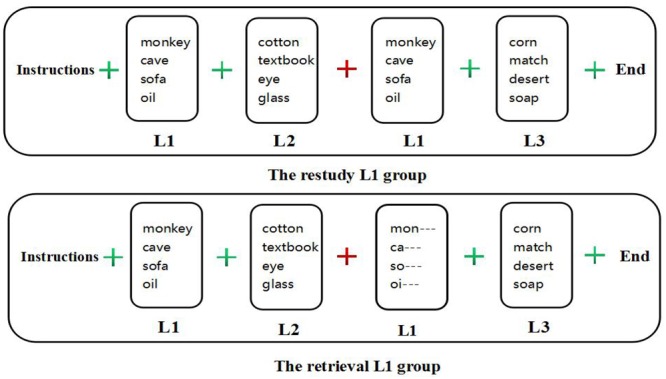
The procedure of Experiment 1.

For the restudy groups, we first presented the following instructions on the screen: “This experiment aims to study our memory. It is divided into three lists that you must memorize. Each list is separated by a plus sign ‘+’; a green plus means ‘Continue the memory task,’ and a red plus means ‘Please restudy L1.’ When you are ready, press the space bar to begin.” Then, we presented L1, a green “+” for 1 s, L2, a red “+” for 1 s, L1, and L3. Finally, we presented the instruction: “The experiment is over.” Then, the participants first completed Task 1 and then Task 2 (Figure 2).

Before the commencement of the formal experiment, all participants were required to perform the practice experiment to become familiar with the experimental procedures and understand the instructions. In the practice experiment, each list comprised two words, and other procedures were consistent with the formal experiment. The practice experiments were not experimental tasks to ensure that the participants were blinded to the purpose of the experiment.

### Results

#### Task 1

In the 2 memory material (concrete words/abstract words) × 2 memory task (retrieval L1/restudy L1) design, the two-factor complete random analysis of variance showed a significant main effect of memory materials [*F*(1,56) = 4.248, *p* < 0.05, $\eta_{p}^{2}$= 0.071]. The *post hoc* comparison revealed a significant greater number of correct answers in the free recall L2 task in the abstract words group (*M* = 1.8667, *SD* = 0.730) than in the concrete words group (*M* = 1.4667, *SD* = 1.042, *p* < 0.05). The main effect of the memory tasks was not significant [*F*(1,56) = 3.121, *p* > 0.05]. The interaction effect was not significant [*F*(1,56) = 0.780, *p* < 0.05, $\eta_{p}^{2}$= 0.014] (Table 1).

**Table 1:** Analysis of variance in Task 1 (Experiment 1).

Source	*SS*	*df*	*MS*	*F*	*p*
Memory materials	3.267	1	3.267	4.248	0.044
Memory tasks	2.400	1	2.400	3.121	0.083
Memory materials × Memory tasks	0.600	1	0.600	0.780	0.381
Error	43.067	56	0.769		

#### Task 2

In the 2 memory material (concrete words/abstract words) × 2 memory task (retrieval L1/restudy L1) design, the two-factor complete random analysis of variance did not reveal significant main effects of memory materials [*F*(1,56) = 0.001, *p* = 0.976] or memory tasks [*F*(1,56) = 0.669, *p* = 0.417]; the interaction effect was not significant [*F*(1,56) = 0.669, *p* = 0.417] (Table 2).

**Table 2:** Analysis of variance in Task 2 (Experiment 1).

Source	*SS*	*df*	*MS*	*F*	*p*
Memory materials	0.017	1	0.017	0.001	0.976
Memory tasks	12.150	1	12.150	0.669	0.417
Memory materials × Memory tasks	12.150	1	12.150	0.669	0.417
Error	1016.667	56	18.155		

## Experiment 2

According to Experiment 1, the explanation for the lack of a significant main effect of the “memory task” may be attributed to two points: (1) each list contained too few words, and (2) the difference in the context change, which was caused by the “retrieval L1 task” and the “restudy L1 task,” was not significant. However, our study focuses on working memory; therefore, changing the word items in each list is not appropriate. If each list contains three or fewer words, the memory items of all experiments will be equal to or less than nine words. At this time, our independent variable will be confused with the differences in the short-term memory abilities of the participants. If each list contains five or more words, the participants’ memory of items will be exhausted after the memory of L2. Therefore, L3 will exist in name only, and the first effect and the recent effect will be more prominent.

Therefore, we should consider changing the “retrieval L1” task. In previous studies using the list-before-last paradigm, when the “memory tasks” variable contained the “retrieval L1” task and the “mathematical problem-solving task” for two levels, the context change caused by these tasks exhibited significant differences, and a significantly greater number of correct answers for free recall L2 was observed in the mathematical problem-solving group (Sahakyan and Hendricks, 2012). Perhaps by significantly reducing the number of retrieval cues for inhibitory information, we will observe a significant difference in context change between the different memory tasks. Therefore, we will replace the “retrieval L1 task” with “free recall L1 task” in Experiment 2, and we expect that different memory tasks, which are based on the presentation of all or no retrieval cues, will produce a significant context change between the different groups.

According to two previous studies (Sahakyan and Hendricks, 2012; Sahakyan and Smith, 2014), the “retrieval L1” group recorded the fewest number of free recall L2 words compared with the “restudy L1” group and the “mathematical problem-solving” group. We do not know which of the latter groups recorded the greatest number of free recall L2 words. However, a greater number of free recall L2 words was recorded in the no-retrieval-clues for inhibitory information condition than in the yes-retrieval-clues for the inhibitory information condition. Therefore, we hypothesize that in Task 1 of Experiment 2, a greater number of free recall L2 words will be recorded by the “free recall L1” group than by the “restudy L1” group. In Task 2, more position change units will be observed for the “restudy L1” group than for the “free recall L1” group.

Additionally, the emotional valence values for all words in Experiment 2 were as follows: concrete word, *M* = 4.2689; and abstract word, *M* = 4.2633. The difference between the two types of words was not significant.

### Methods

#### Participants

We recruited a group of 60 volunteers from Hebei University who ranged in age between 18 and 25 years (*M* = 22.07, *SD* = 2.10), were not color blind and had normal vision or normal corrected vision. In Experiment 2, participants were randomly assigned to the following four experimental groups: the abstract words free recall L1 group; the abstract words restudy L1 group; the concrete words free recall L1 group; and the concrete words restudy L1 group. Each group contained 15 participants. After the experiment, each person was rewarded with a gift (stationery, such as pens and notebooks).

#### Materials

All words were the same as those used in Experiment 1.

#### Design

The study employed a 2 memory material (concrete words/abstract words) × 2 memory task (free recall L1/restudy L1) between-subjects design. Other details were the same as Experiment 1.

#### Procedure

**FIGURE 3:**
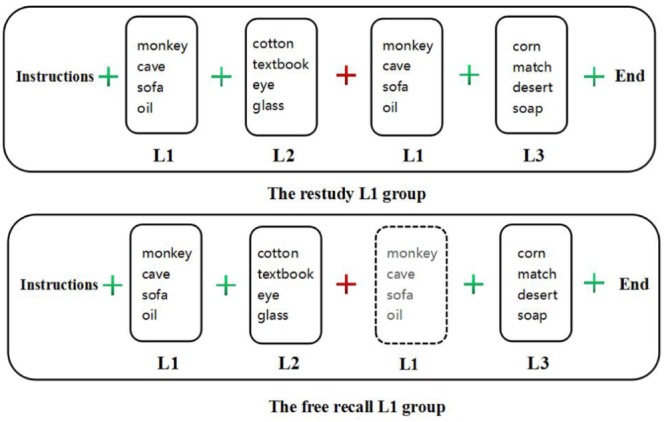
The procedure of Experiment 2.

In the free recall L1 group, the instructions were as follows: “This experiment aims to study our memory. It is divided into three lists that you must memorize. Each list is separated by a plus sign ‘+’; a green plus means ‘Continue the memory task,’ and a red plus means ‘Please keep looking at the red plus and memorize L1.’ When you are ready, press the space bar to begin.” Other details were the same as in Experiment 1 (Figure 3).

### Results

#### Task 1

**FIGURE 4:**
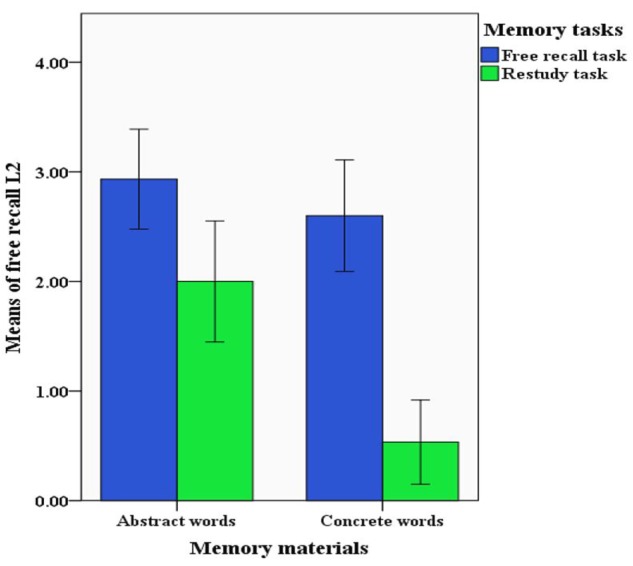
The interaction effect between memory materials and memory tasks (Experiment 2).

In the 2 memory material (concrete words/abstract words) × 2 memory tasks (free recall L1/restudy L1) design, the two-factor complete random analysis of variance showed a significant main effect of memory materials [*F*(1,56) = 14.097, *p* < 0.05, $\eta_{p}^{2}$ = 0.201]. The *post hoc* comparison showed a greater number of correct answers for free recall L2 in the abstract words group (*M* = 2.467, *SD* = 1.074) than in the concrete words group (*M* = 1.567, *SD* = 1.357, *p* < 0.05). The main effect of memory tasks was significant [*F*(1,56) = 39.157, *p* < 0.05, $\eta_{p}^{2}$ = 0.412]. The *post hoc* comparison showed a significantly greater number of correct answers for free recall L2 in the free recall group (*M* = 2.767, *SD* = 0.935) than in the restudy group (*M* = 1.267, *SD* = 1.172, *p* < 0.05). The interaction effect was significant [*F*(1,56) = 5.588, *p* < 0.05, $\eta_{p}^{2}$ = 0.091] (Table 3). The simple effect analysis showed that the participants restudying L1 in the abstract words recorded a significantly greater number of correct responses for free recall L2 (*M* = 2.000, *SD* = 1.069) than the participants restudying L1 in the concrete words (*M* = 0.533, *SD* = 0.743, *p* < 0.05) (Figure 4).

**Table 3:** Analysis of variance in Task 1 (Experiment 2).

Source	*SS*	*df*	*MS*	*F*	*p*
Memory materials	12.150	1	12.150	14.097	0.000
Memory tasks	33.750	1	33.750	39.157	0.000
Memory materials × Memory tasks	4.817	1	4.817	5.588	0.022
Error	48.267	56	0.862		

#### Task 2

In the 2 memory material (concrete words/abstract words) × 2 memory task (free recall L1/restudy L1) design, the two-factor complete random analysis of variance revealed a significant main effect of memory materials [*F*(1,56) = 11.677, *p* < 0.05, $\eta_{p}^{2}$= 0.173]. The *post hoc* comparison showed a significantly greater number of position change units of L2 in the concrete words group (*M* = 7.300, *SD* = 4.893) than in the abstract words group (*M* = 5.033, *SD* = 4.056, *p* < 0.05). The main effect of memory tasks was significant [*F*(1,56) = 117.818, *p* < 0.05, $\eta_{p}^{2}$= 0.678]. The *post hoc* comparison showed a significantly greater number of position change units of L2 in the restudy L1 group (*M* = 9.767, *SD* = 3.266) than in the free recall L1 group (*M* = 2.567, *SD* = 2.359, *p* < 0.05). The interaction effect was not significant [*F*(1,56) = 3.646, *p* = 0.061] (Table 4).

**Table 4:** Analysis of variance in Task 2 (Experiment 2).

Source	*SS*	*df*	*MS*	*F*	*p*
Memory materials	77.067	1	77.067	11.677	0.001
Memory tasks	777.600	1	777.600	117.818	0.000
Memory materials × Memory tasks	24.067	1	24.067	3.646	0.061
Error	369.600	56	6.600		

## General Discussion

The present study found the cognitive inhibitory effect on working memory was influenced by the nature of the processed information and the number of inhibitory information retrieval cues.

In Experiment 1, both tasks, we didn’t examine a significant difference between retrieval L1 task and restudy L1 task as the previous studies did. And we hypothesized that the number of fewer items in each list may had contributed to this result compared with previous studies. However, we examined a significant difference between concrete words and abstract words in Task 1. This finding was consist with the concreteness effect theory.

And in Experiment 2, both tasks observed the concreteness effect and cognitive inhibitory effect during the information retrieval phase. Especially in Task 2, the results indicated by our new statistical indicator “the amount of position change units in L2” was consist with Task 1 results, this finding further proved that the position change amount of inhibition information may be used as a statistical standard of individual’s context change.

### Concreteness Effects of Cognitive Inhibition on Working Memory

The concreteness effect may also exist in the information retrieval phase. In our study, a greater number of correct answers for the free recall of L2 abstract words was observed compared with concrete words. According to a previous study, the processing of concrete words in the information processing phase may activate more semantic information than the processing of abstract words (Kounios and Holcomb, 1994). In other words, a greater context change will occur when we process concrete words. Therefore, when we experimentally controlled the information retrieval time and mode, we observed a smaller cognitive inhibition effect during the concrete words processing procedure than in the abstract words processing procedure.

Clearly, the information we process in our daily life is more concrete, and the cognitive inhibitory effect on these types of information will thus be greater. For example, if we were asked at noon, “Did you eat in the morning?” we may easily answer this question, but if we were asked, “What did you eat in the morning? What did you eat first and what did you eat afterwards?” we may need to think for a while. Moreover, the concreteness effect not only exists in the information processing phase but also in the information retrieval phase; it can cause difficulty in retrieving information our daily life. Namely, if we specifically remember one thing, we may completely forget another concrete thing, and after a period of time, with the degree of the information’s concreteness decreasing naturally, the inhibited information may be easier for us to retrieve as the degree of the concreteness of the information decreases naturally. However, the information will become more confusing, and its summary may even be wrong at this later time point.

### Relationship Between the Number of Inhibitory Information Retrieval Cues and the Cognitive Inhibitory Effect

The result in Experiment 1 differs from a previous study in which the number of correct answers for free recall L2 were greater in the restudy group than in the retrieval group (Sahakyan and Smith, 2014). This discrepancy may be caused by the difference in the number of items in each list. In the previous study, each list contained twelve words. Considering the short-term memory span (7 ± 2 units) and “the first four words effect” as we already have said (Pastötter et al., 2012), we used four words in each list in this study. That is to say, whether inhibitory information retrieval cues were processing partly or totally, the effect of cognitive inhibition on working memory may not be obvious with less memory load. And only when the working memory load reaches a certain amount (more than four), the differential amount of inhibitory information retrieval cues will cause a significant cognitive inhibition effect. Additionally, The lack of a significant main effect of memory tasks performed in Experiment 1 may also indicate that the cognitive inhibitory effect was not affected by the number of retrieval cues for inhibitory information; at least, the effect is not significant. But, in Experiment 2, under two extreme conditions in which the retrieval cues for inhibitory information were all provided or no cues were provided, a greater cognitive inhibitory effect was observed for the former condition. However, the relationship between the number of inhibitory information retrieval cues and the cognitive inhibitory effect may not be linear because previous studies have reported that the presentation of inhibitory information retrieval cues actually resulted in the greatest cognitive inhibitory effect (Sahakyan and Hendricks, 2012; Sahakyan and Smith, 2014). This finding is interesting.

The study also found that using an “all or none” classification method for inhibitory information retrieval cues. The number of retrieval cues for inhibitory information may lead to different levels of cognitive inhibitory effect, even with a less memory load. Accordingly, different levels of cognitive inhibitory effect can be reflected through the number of position change units of inhibition information. In Experiment 2, two tasks’ results were consistent with each other and partially confirmed our earlier hypothesis that in the list-before-last paradigm when all retrieval cues for the inhibitory information are presented, a greater cognitive inhibitory effect will be observed than for the inhibitory information with no retrieval cues. Furthermore, participants recalled most of the L1 words in two experiments. However, when we ignored the number of L1 words participants retrieved during the final test, participants actually retrieved all 4 words in L1 under both conditions; therefore, the effect of L1 differed because of the different processing pathways. According to the results of Experiment 2, if participants retrieved L1 words through the use of all retrieval cues, then the inhibitory effect of L1 will be greater than another pathway in which L1 words were retrieved with no retrieval cues. Thus, with the exception of the effect of the amount of inhibitory information on the extent of the inhibitory effect, the pathways used to retrieve inhibitory information may also be effective.

A high-level cognitive inhibitory effect based on all amount of inhibited information cues was more likely to occur in the information processing of low cognitive resource consumption. According to context change model, the greatest number of retrieval cues for concrete words condition produced a greater context change effect (inhibitory effect) that transferred to the other homogeneous concrete words than the fewest (none) number of retrieval cues condition. As shown in previous studies, the “concreteness effect” is present when individuals are processing information (James, 1975; Schwanenflugel and Shoben, 1983; Kroll and Merves, 1986; Bleasdale, 1987; Schwanenflugel et al., 1988). However, based on our findings, differences in degree of the activated semantic information between abstract words and concrete words may also have existed when individuals retrieved information. According to the TCM, the represented concrete information may activate more semantic changes and cause greater changes in the memory of the order of the other homogeneous concrete information. Unfortunately, we did not observe a significant interaction effect in Task 2. We must reconsider the rationality of the dependent variable and its statistical indicator in Task 2.

Moreover, in our experimental memory task, inhibitory information was not irrelevant information about the memory task goal. In fact, regardless of whether inhibitory information (L1) or inhibited information (L2) was related to the completion of the experimental memory task, the information belonged to the same information processing sequence and the same cognitive inhibitory situation in the experiments. Although we did not count the number of participants’ free recall L1 words, participants were able to correctly recall most of the words in L1 when they performed Task 1 in each experiment. Therefore, in the list-before-last paradigm, the method in which we considered L1 as the inhibitory information was somewhat reasonable.

### Mechanism of the Cognitive Inhibitory Effect on Working Memory

The mechanism of this “turn around and forget” phenomenon, which was considered a cognitive inhibition phenomenon in our study, may theoretically consist of the context change model and may practically operate under the TCM. Because we observed a significant retrieval position change in L2 words in the retrieval phase of Experiment 2, the information retrieval sequence had already been affected by the cognitive inhibitory effect, in which the retrieval of the latter information was limited by the retrieval of the former information.

Notably, the observation of a greater context change does not mean that the individual experienced difficulty in processing or retrieving information. Although a difference in the difficulty of memory between the two levels of independent variable (memory tasks), which are distinguished by the number of the retrieval cues for inhibitory information, this type of difference does not represent evidence of the ability to distinguish the two levels of independent variables. Furthermore, the essence of independent variable in the two levels of memory tasks is the difference in the number of inhibitory information retrieval cues, and the essence of the difference in the number of inhibitory information retrieval cues is the difference in the cognitive inhibitory modes. The cognitive inhibition situations, which are caused by the different cognitive inhibitory modes, represent the crux of the list-before-last paradigm. This hypothesis enables the list-before-last paradigm to prove the context change model.

### Applicability of New Statistical Indicators of Context Change and Limitations of the Experiment

Using the number of position change units as a statistical indicator of internal context change is a open question. In our two experiments, the results from Tasks 1 and 2 were not completely matched in each experiment; therefore, we were not able to definitively conclude that the position change of inhibited information (L2) in the retrieval phase represents a statistical indicator of context change. We should consider the limitations of our experiments to explore the reasons for the inconsistent results.

On one hand, although the entire experiment was performed in 5 min, we were not able to avoid individual differences in normal forgetting in our experiments; therefore, the retrieval sequence of each word may not be equivalent when the participants retrieved the individual words in Task 2. We asked the participants to complete Tasks 1 and 2 separately in our experiments to ensure efficiency. However, according to the TCM (Sahakyan and Hendricks, 2012), the retrieval of the former information can influence the retrieval of the latter information. In our experiments, the participants’ memory of concrete information in Task 1 may have affected their retrieval procedure in Task 2, and this experimental error was not controlled for in the experiments.

On the other hand, we controlled for participants’ working memory abilities regarding the individual differences before the experiments, and previous studies confirmed that our methodology was appropriate (Klein and Fiss, 1999; Conway et al., 2005). However, the control measure that uses the participants’ working memory span to represent their working memory capacity might still cause experimental error. In particular, in Task 2, our participants found it challenging to memorize all twelve words. Moreover, in a previous study (Buczny et al., 2015), the participants’ cognitive inhibition types and the loss of cognitive inhibition caused an implicit attitude change toward the same tasks. Specifically, the “directed cognitive inhibition type” (individuals who prefer to inhibit the irrelevant concrete information when completing a task) participants may have an advantage in completing the task than the “undifferentiated cognitive inhibition type” (individuals who prefer to inhibit all processed information when completing a task) participants. Simultaneously, the natural loss of the cognitive inhibition of the participants over time will have a certain effect on target completion. Therefore, these factors that were considered a source of systematic error in our experiments that we were not able to control, and thus may also be a source of experimental error in our studies.

### Research Development and Prospects

The use of Chinese materials as experimental materials in our study might be feasible under the condition that distinguished Chinese words by their different nature, and we observed a significant concreteness effect in our experiments. However, we still do not know the applicability of other types of Chinese experimental materials, such as the Chinese adjectives (which may relate to an individual’s emotion and motivation) or Chinese verbs (which may relate to an individual’s embodied cognition), which may also affect information processing and cognitive inhibition in the working memory task using the list-before-last paradigm. On this issue, future research needs to be further refined.

Furthermore, considering the item numbers of each list when researcher transform the list-before-last paradigm seems to be necessary. The most obvious difference compared with original study was “the number of items in each list” and we suspect that it was the main reason for the inconsistency results. Additionally, when participants freely recalled L2 in the final test, recent research observed significant differences in L3 intrusions between the math group (using a distraction task between L2 and L3 in list-before-last paradigm) and the retrieval L1 group (Sahakyan and Hendricks, 2012). The math group had significantly more intrusions than the retrieval group. However, in our study, particularly in Experiment 2, we created a free recall L1 group that was similar to the math group, but we still did not observe significant L3 intrusions in the final free recall L2 test.

In addition to the number of position change units, time estimates have also been shown to represent a marker of internal context change (Sahakyan and Smith, 2014). In terms of verbal estimates, the retrieval group recorded significantly longer time estimates throughout the experiment than the restudy group in the list-before-last paradigm, although the duration of the experiment was equal in both groups. Although this finding has not been confirmed by a sufficient number of studies, it might still represent a reference marker of internal context change.

In addition to experimental materials and experimental paradigms, future research can also focus on the methods used to present experimental materials due to the current rapid development of augmented reality (AR) and virtual reality (VR) technology. The perception of different spatial scales (particularly the large-scale space) by individuals might also affect the mechanism of the cognitive inhibitory effect on working memory. The application of additional neural science technology might be useful in investigations of the mechanism underlying the cognitive inhibitory effect on working memory at the technical level.

## Ethics Statement

The authors have read and approved the submission of the manuscript. It has not been published in this or a substantially similar form (in print or electronically, including on a web site), nor accepted for publication or consideration thereof elsewhere, in whole or in part, in any language. The study was approved by the academic and ethics committee of school of education in Hebei University. The academic and ethics committees approved this consent procedure. All procedures performed in studies involving human participants were in accordance with the ethical standards of the institutional and/or national research committee and with the 1964 Helsinki declaration and its later amendments or comparable ethical standards. Informed consent was obtained from all individual participants included in the study.

## Author Contributions

XZ, CL, and CS conceived and designed the study. XZ and CL performed the study. XZ and CS analyzed the data. XZ, CL, and CS wrote the paper.

## Conflict of Interest Statement

The authors declare that the research was conducted in the absence of any commercial or financial relationships that could be construed as a potential conflict of interest.
